# Viewing the body modulates both pain sensations and pain responses

**DOI:** 10.1007/s00221-016-4585-9

**Published:** 2016-02-16

**Authors:** Brianna Beck, Elisabetta Làdavas, Patrick Haggard

**Affiliations:** Centre for Studies and Research in Cognitive Neuroscience, University of Bologna, Viale Europa 980, 47023 Cesena, Italy; Department of Psychology, University of Bologna, Viale Berti Pichat 5, 40127 Bologna, Italy; Institute of Cognitive Neuroscience, University College London, 17 Queen Square, London, WC1N 3AZ UK

**Keywords:** Crossmodal, Body, Nociception, Pain, Signal detection

## Abstract

Viewing the body can influence pain perception, even when vision is non-informative about the noxious stimulus. Prior studies used either continuous pain rating scales or pain detection thresholds, which cannot distinguish whether viewing the body changes the discriminability of noxious heat intensities or merely shifts reported pain levels. In Experiment 1, participants discriminated two intensities of heat-pain stimulation. Noxious stimuli were delivered to the hand in darkness immediately after participants viewed either their own hand or a non-body object appearing in the same location. The visual condition varied randomly between trials. Discriminability of the noxious heat intensities (*d*′) was lower after viewing the hand than after viewing the object, indicating that viewing the hand reduced the information about stimulus intensity available within the nociceptive system. In Experiment 2, the hand and the object were presented in separate blocks of trials. Viewing the hand shifted perceived pain levels irrespective of actual stimulus intensity, biasing responses toward ‘high pain’ judgments. In Experiment 3, participants saw the noxious stimulus as it approached and touched their hand or the object. Seeing the pain-inducing event counteracted the reduction in discriminability found when viewing the hand alone. These findings show that viewing the body can affect both perceptual processing of pain and responses to pain, depending on the visual context. Many factors modulate pain; our study highlights the importance of distinguishing modulations of perceptual processing from modulations of response bias.

## Introduction

Pain provides important information about the state of the body, as well as external objects that threaten the body. The sensation of noxious heat on the skin is an important experimental model of pain. It depends on activation of nociceptive afferents that project to the brain via the spinothalamic pathway (Willis et al. 1979). Centrally, this nociceptive input may interact with other senses that convey information about the body, including touch (Inui et al. 2006; Mancini et al. 2014; Mouraux and Plaghki 2007) and vestibular sensation (Ferrè et al. 2013). Noxious stimuli also reduce corticospinal excitability, indicating a central inhibitory effect of pain on the motor system (Farina et al. 2003; Le Pera et al. 2001). These interactions between pain, innocuous sensation, and motor function may contribute to a multimodal representation of the body that facilitates responses to potentially injurious events (Haggard et al. 2013).

Pain perception is also modulated by sensory modalities such as vision that are not somatic per se, but provide a context for pain perception. Viewing the body can reduce the perceived intensity of a painful stimulus (Longo et al. 2009, 2012; Mancini et al. 2012, 2013; Valentini et al. 2015,1) and increase pain detection thresholds (Mancini et al. 2011) relative to viewing a non-body object. Viewing the body also reduces the amplitude of noxious laser stimulus-evoked potentials (LEPs; Longo et al. 2009) and alters beta oscillations over sensorimotor cortex (Mancini et al. 2013). Additionally, viewing the body during painful stimulation increases functional connectivity between posterior parietal areas that process visual body information and the putative ‘pain matrix’—primary (SI) and secondary (SII) somatosensory cortex, the anterior and posterior insula, and anterior cingulate cortex (Longo et al. 2012).

While previous studies showed that viewing the body affects pain, it is unclear whether the changes relate to nociceptive processing specifically or to post-perceptual cognitive functions. The pain matrix, despite its name, is composed of several nodes that perform various functions, some of which are not specific to pain, such as arousal and threat detection (Cauda et al. 2012; Hayes and Northoff 2012; Iannetti and Mouraux 2010; Legrain et al. 2011; Lötsch et al. 2012; Mouraux et al. 2011). Similarly, LEPs may reflect a domain-general measure of stimulus salience rather than pain sensation in particular (Iannetti et al. 2008; Mouraux and Iannetti 2009). Thus, it is unclear whether viewing the body results in a functional loss of information from the nociceptive system, or changes responses to pain.

Using signal detection theory (Green and Swets 1966), we investigated whether viewing the body reduces the discriminability of noxious heat stimulation levels (i.e., a loss of information about stimulus intensity) or induces a bias in perceived pain level (i.e., a non-discriminative effect in which the probability of responding ‘high pain’ is changed, irrespective of the actual stimulus intensity). This distinction was difficult to make in previous studies because pain perception was measured using continuous pain rating scales (Longo et al. 2009, 2012; Mancini et al. 2012, 2013; Valentini et al. 2015) or pain detection thresholds (Mancini et al. 2011). Instead, we used binary forced choice pain intensity judgments to obtain separate measures of discriminability and response bias (Lockwood et al. 2013; Mancini et al. 2014). Our approach differed from earlier applications of signal detection theory to pain in that it required participants to discriminate a higher and a lower level of painful stimulation, rather than rating both painful and non-painful stimulation levels (see Rollman 1977 for a review). Thus, we specifically examined perceptual processing *within* nociceptive pathways, as opposed to *between* noxious and non-noxious stimuli.

While several studies have investigated the effects of viewing the body on pain perception, the visual stimuli used vary substantially [e.g., one’s own hand (Longo et al. 2009, 2012; Mancini et al. 2012, 2013, 2011; Valentini et al. 2015), another’s hand (Longo et al. 2009), or another’s hand perceived as one’s own (Höfle et al. 2012, 2013)]. In addition, some studies displayed a pain-inducing event on the body (Höfle et al. 2012, 2013; Mancini et al. 2013). In one such study, viewing an image of a needle pricking a hand increased pain ratings of intracutaneous electrical stimuli (Höfle et al. 2012, 2013). In another study, however, participants gave lower pain ratings when viewing a thermode probe deliver a painful heat stimulus to their hand compared to viewing the probe touch a non-body object (Mancini et al. 2013). Thus, there is contradictory evidence for whether viewing a threatening stimulus approach the body has a similar effect on pain perception as viewing the body alone. We systematically compared these two conditions and their effects on both discriminability of noxious stimulus intensities and biases in perceived pain level.

## General method

### Participants

Separate groups of 16 volunteers were recruited for each of the three experiments (Experiment 1: 10 female, *M*_age_ = 26.63 years, SD_age_ = 8.01; Experiment 2: 6 female, *M*_age_ = 27.31 years, SD_age_ = 8.92; Experiment 3: 10 female, *M*_age_ = 23.81 years, SD_age_ = 5.08). Two participants in Experiment 2 and one in Experiment 3 were excused during pain threshold determination and subsequently replaced because they did not perceive even the highest safe level of thermal stimulation as painful. The study was approved by the University College London Research Ethics Committee. Informed consent was obtained from all participants. All procedures performed were in accordance with the ethical standards of the institutional research committee and with the 1964 Helsinki Declaration and its later amendments.

### Materials

**Fig. 1:**
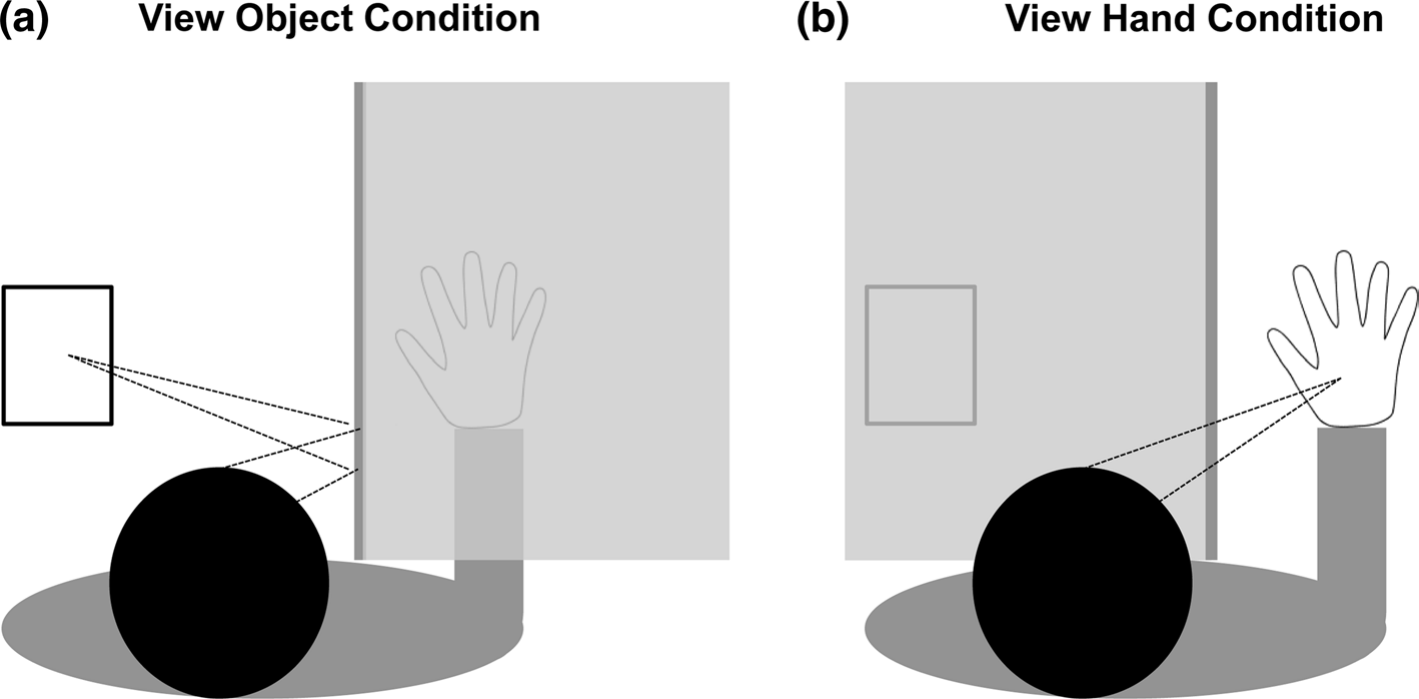
Experimental setup with semi-silvered mirror. **a** When the *left* side of the mirror was illuminated, the foam block was seen in place of the *right* hand. **b** When the *right* side of the mirror was illuminated, the *right* hand was seen in its true location

Contact heat-pain stimuli were delivered with a Peltier thermode connected to a 13-mm-diameter pen-shaped probe (Physitemp NTE-2A, Clifton, NJ). The probe was attached to a wood bar controlled by a high-power servo motor (Hitec HS-805BB, Poway, CA) that brought the tip into contact with the hand dorsum. Visual presentation was controlled by a semi-silvered mirror embedded in a barrier with a light-emitting diode (LED) lamp on each side. Participants placed their right hand to the right of the mirror. A hand-sized foam block was situated to the left of the mirror behind another barrier that prevented direct vision of it. When the lamp on the left side was illuminated, participants saw the reflection of the foam block, so that it appeared in the same location as their right hand (Fig. 1a). When the lamp on the right side was illuminated, participants instead saw their right hand through the mirror (Fig. 1b).

### Experiment 1

#### Procedure

First, each participant’s pain threshold was determined. The thermode probe was placed on the right hand dorsum. Beginning at 32 °C, the probe temperature increased at 0.5 °C/s until the participant pressed a button with the left hand to indicate that the heat had just begun to elicit a painful, pinprick-like sensation. This temperature ramp was done four times, and the average temperature at which the participant pressed the button was taken as the pain threshold. To avoid peripheral effects on pain perception such as receptor adaptation, vascular responses, and persistent changes in skin temperature, the right hand was moved slightly between ramps.

The pain threshold was used to set a medium level of heat pain (approx. 2 °C above threshold) and a high level of heat pain (approx. 4 °C above threshold). Participants completed two practice blocks of ten trials each in which they distinguished medium and high heat-pain stimuli. Each thermal stimulus was 1 s long, with a 12-s interstimulus interval. The thermal stimulus was preceded by a ramp up to the target temperature during the interstimulus interval, when the thermode was not in contact with the skin. The hand was moved slightly between blocks. To avoid floor and ceiling effects, the high heat-pain stimulus was adjusted in increments of 1 °C if participants answered fewer than 65 % or more than 85 % of trials correctly. Likewise, the medium-pain stimulus was increased by 1 °C if it was not consistently perceived as painful. For safety reasons, thermal stimulation never exceeded 50 °C.

Once the medium and high heat-pain levels were set, participants completed eight experimental blocks. Each block contained six medium and six high heat-pain trials presented in a random order and equiprobably with the hand and object visual conditions. The experiment was carried out in a dark room for visual stimulus control. On each trial, a lamp turned on for 2 s, revealing either the participant’s hand or the foam block. Immediately after lamp offset, the thermode probe descended (0.5 s), touched the back of the hand (1 s), and retracted (0.5 s). Note that participants did not see the thermode probe approach or touch their hand in this experiment. Participants pressed a button with their left hand to indicate whether they felt a medium or high heat-pain stimulus. To minimize peripheral effects on pain perception, the right hand was moved slightly between blocks, and the intertrial interval was 9 s.

#### Results

The high heat-pain level was arbitrarily defined as the target. A ‘hit’ was thus a high heat-pain stimulus identified as ‘high,’ while a ‘false alarm’ was a medium heat-pain stimulus identified as ‘high.’ Proportions of hits and false alarms were used to calculate measures of discriminability (*d*′) and response bias (criterion; Green and Swets 1966) for each individual participant in each visual condition (hand or object), according to the following equations:

1 $$d^{\prime} = z\left( {\text{hit}\,\text{rate}} \right) - z\left( {\text{false}\,\text{alarm}\,\text{rate}} \right)$$

2 $$\text{Criterion} = - 0.5*\left[ {z\left( {\text{hit}\,\text{rate}} \right) + z\left( {{\text{false}}\,\text{alarm}\,\text{rate}} \right)} \right]$$

**Fig. 2:**
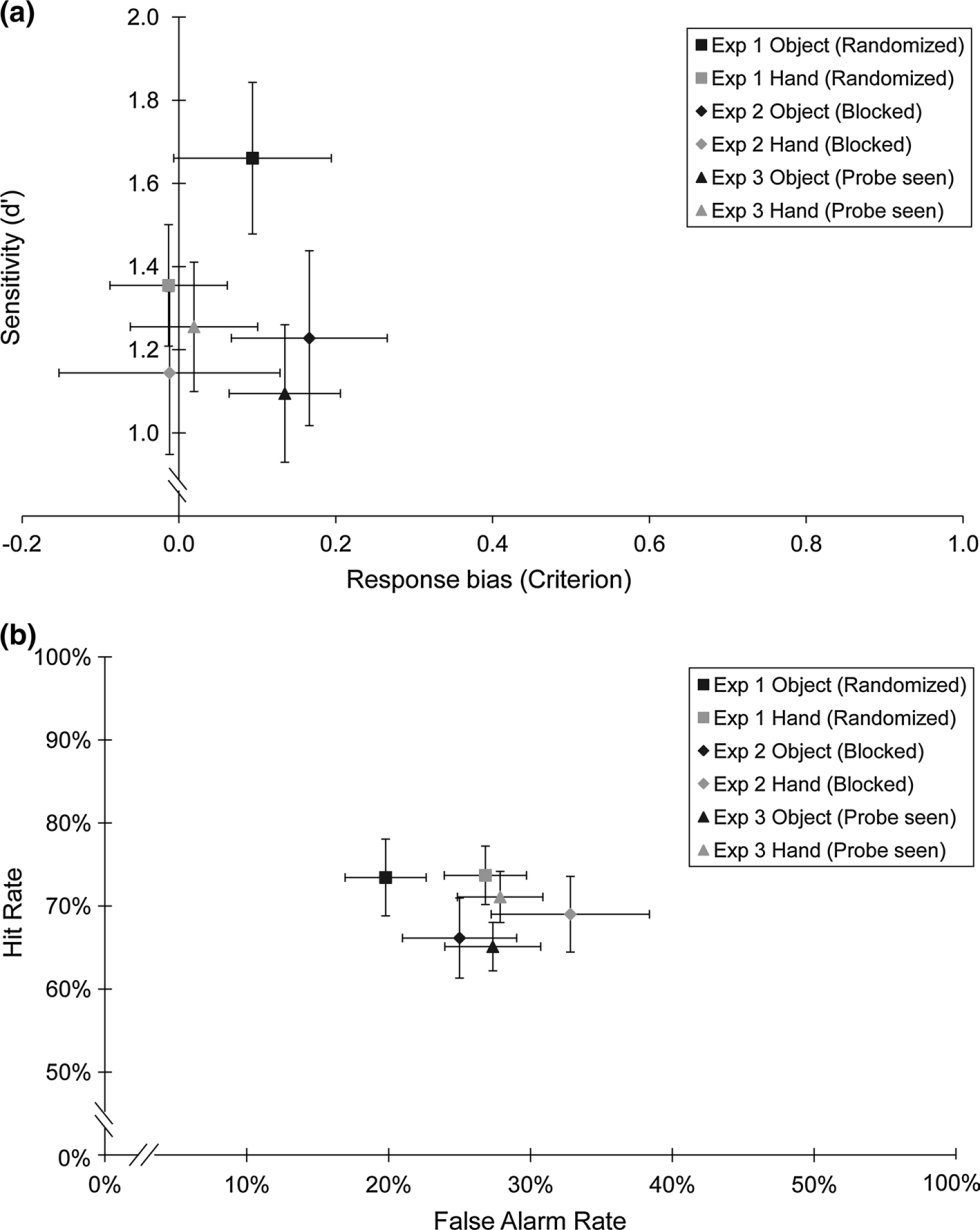
Results of Experiment 1 (randomized presentation of hand/object), Experiment 2 (blocked presentation of hand/object), and Experiment 3 (probe seen approaching hand/object). **a** Mean (±SEM) scores of discriminability (*d*′) on the *y* axis and response bias (criterion) on the *x* axis. An increase on the *y* axis indicates enhanced discriminability of noxious heat intensities. An increase on the *x* axis indicates a greater tendency to respond ‘medium,’ irrespective of actual stimulus intensity. **b** Mean (±SEM) hit rates (percentages of high heat-pain stimuli called ‘high’) on the *y* axis and false alarm rates (percentages of medium heat-pain stimuli called ‘high’) on the *x* axis. An increase on the *y* axis indicates a higher proportion of high heat-pain stimuli perceived as ‘high.’ An increase on the *x* axis indicates a higher proportion of medium heat-pain stimuli perceived as ‘high’

Measures of *d*′ and criterion for each participant were then entered into statistical analyses. Paired-samples *t* tests compared *d*′ and criterion scores in the hand and object visual conditions. Discriminability (*d*′) was lower after participants saw their hand (*M* = 1.35, SD = 0.58) than after they saw the object (*M* = 1.66, SD = 0.73), *t*(15) = 2.24, *p* = .041, Cohen’s *d* = .470. There was no difference in bias (criterion) between hand (*M* = −0.01, SD = 0.30) and object (*M* = 0.09, SD = 0.40) conditions, *t*(15) = 1.22, *p* = .243, Cohen’s *d* = .283. This indicates that viewing the body reduced the discriminability of noxious stimulation levels rather than biasing pain responses (Fig. 2a).

To see whether the reduction in discriminability after viewing the hand had an analgesic effect, as previous studies have found (Longo et al. 2009, 2012; Mancini et al. 2011, 2012, 2013), we compared proportions of hits and false alarms after participants viewed the hand and the object. Because the target stimulus was the high heat-pain level, a lower hit rate would indicate a decrease in perceived heat-pain intensity. In fact, there was no difference in the hit rate between the hand condition (*M* = 73.70 %, SD = 14.09 %) and the object condition (*M* = 73.44 %, SD = 18.50 %), *t*(15) = −0.08, *p* = .936, Cohen’s *d* = −.016. Instead, participants made more false alarms after viewing their hand (*M* = 26.82 %, SD = 11.58 %) than after viewing the object (*M* = 19.79 %, SD = 11.44 %), *t*(15) = −2.30, *p* = .036, Cohen’s *d* = −.611, meaning that more medium heat-pain stimuli were perceived as high. In contrast to previous studies, viewing the hand yielded an *increase* in perceived heat-pain intensity at lower levels of noxious stimulation (Fig. 2b).

#### Discussion

Experiment 1 demonstrated that viewing the body modulates pain perception at a perceptual level, rendering intensities of noxious stimulation less discriminable. Surprisingly, this reduction in discriminability resulted in more medium heat-pain stimuli being perceived as high. Previous studies that used continuous rating scales or pain detection thresholds instead found that viewing the body decreases perceived pain levels (Longo et al. 2009, 2012; Mancini et al. 2011, 2012, 2013; Valentini et al. 2015). These measures differ from the binary forced choice task we used in that they do not require participants to discriminate different intensities of noxious stimulation. Differences between task sets may have led to a change in the direction of the effect of viewing the body on perceived pain level.

Alternatively, differences in visual stimulation between Experiment 1 and previous studies might account for the discrepant findings. Earlier studies (Longo et al. 2009, 2012; Mancini et al. 2011, 2012, 2013; Valentini et al. 2015) presented the hand and object visual conditions in separate blocks [except the second experiment reported by Mancini et al. (2013), which yielded a markedly smaller reduction in perceived pain intensity when viewing the hand]. In Experiment 1, the hand and object visual conditions varied randomly trial-by-trial. Visual exposure was brief, and its content was unpredictable, which may have changed its effect on pain perception. Indeed, predictable painful stimuli are typically perceived as less intense than physically identical but unpredictable painful stimuli (Carlsson et al. 2006; Crombez et al. 1994; Meulders et al. 2012). This effect may generalize beyond the predictability of the painful stimulus of interest to the context in which it is presented (Rhudy and Meagher 2000). Experiment 2 tested this hypothesis by presenting blocks in which participants saw only the hand or the object, making the content of visual stimulation predictable and consistent over time.

### Experiment 2

#### Procedure

The procedure of Experiment 2 was exactly the same as that of Experiment 1, except that participants saw their hand on every trial in half the blocks and the object on every trial in the other half. Block order was counterbalanced (HHOOHHOO for half the participants and OOHHOOHH for the other half). The thermode probe approached and touched the hand in darkness, so participants did not see the pain-inducing event.

#### Results

As in Experiment 1, the high heat-pain level was defined as the target stimulus. One participant did not have any false alarms in the hand visual condition, so a standard correction was applied (Macmillan and Kaplan 1985). The false alarm rate was set to 1/(2 N), where N is the maximum number of false alarms the participant could make (i.e., the total number of medium heat-pain trials in the hand visual condition). This yields a rate halfway between 0 and the smallest false alarm rate the participant could have had, given the number of medium heat-pain trials.

Mixed factor analyses of variance (ANOVAs) with the within-subjects factor ‘visual condition’ (hand or object) and the between-subjects factor ‘experiment’ (visual presentation randomized—Experiment 1—or blocked—Experiment 2) was carried out on *d*′ and criterion scores. There was a trend toward lower *d*′ scores after viewing the hand (*M* = 1.25, SD = 0.69) than after viewing the object (*M* = 1.44, SD = 0.80) regardless of whether they were presented in a randomized or a blocked order, *F*(1,30) = 3.26, *p* = .081, *η*^2^ = .095. There was also a main effect of visual condition on criterion scores, *F*(1,30) = 5.78, *p* = .023, *η*^2^ = .160. Criterion was lower after viewing the hand (*M* = −0.01, SD = 0.44) than after viewing the object (*M* = 0.13, SD = 0.40), meaning that participants were more likely to respond ‘high’ after seeing their hand, irrespective of the actual intensity of the noxious stimulus (Fig. 2a). The ANOVAs further showed no main effects of experiment [*d*′: *F*(1,30) = 1.82, *p* = .188, *η*^2^ = .057; criterion: *F*(1,30) = 0.07, *p* = .793, *η*^2^ = .002], nor interactions between experiment and visual condition [*d*′: *F*(1,30) = 1.06, *p* = .310, *η*^2^ = .031; criterion: *F*(1,30) = 0.36, *p* = .552, *η*^2^ = .010].

Mixed factors ANOVAs on hit and false alarm rates confirmed the results of Experiment 1 alone. There was a main effect of visual condition on the false alarm rate, *F*(1,30) = 10.91, *p* = .002, *η*^2^ = .266. Participants perceived more medium heat-pain stimuli as high heat-pain stimuli after viewing the hand (*M* = 29.82 %, SD = 17.74 %) than after viewing the object (*M* = 22.40 %, SD = 13.99 %) regardless of whether visual presentation was randomized or blocked. There was no main effect of experiment (randomized or blocked presentation), *F*(1,30) = 1.17, *p* = .289, *η*^2^ = .037, and no interaction between experiment and visual condition, *F*(1,30) = 0.03, *p* = .863, *η*^2^ = .001. The hit rate analysis found neither a main effect of visual condition, *F*(1,30) = 0.43, *p* = .515, *η*^2^ = .014, nor of experiment, *F*(1,30) = 1.08, *p* = .308, *η*^2^ = .035. Moreover, there was no interaction between the two factors, *F*(1,30) = 0.30, *p* = .587, *η*^2^ = .010 (Fig. 2b).

#### Discussion

The combined analysis of Experiments 1 and 2 extends the finding that viewing the body yields a functional loss of information about noxious stimulus intensity. In addition to the reduction in discriminability, participants were biased toward reporting a higher level of heat pain after viewing their hand than after viewing the non-body object, regardless of the actual intensity of the noxious heat stimulus. This indicates two potential mechanisms whereby viewing the body might influence pain perception. First, viewing the body has an effect at the sensory level, reducing the discriminability of noxious stimulation intensities. Second, viewing the body biases participants’ criterion for what is painful, in this case leading them to report higher pain levels. We can therefore conclude that the unpredictability of visual stimulation in Experiment 1 was not responsible for the higher perceived pain levels after viewing their hand, because this effect was also present in Experiment 2.

Because there were no interactions between experiment and visual condition, the effects of viewing the body on discriminability and bias manifested to some extent both when the order of hand and object presentation was randomized (Experiment 1) and when the two were presented in separate blocks (Experiment 2). Nevertheless, the reduction in discriminability seems to be stronger in Experiment 1. This effect only reached the level of a trend in the combined analysis of Experiments 1 and 2. Conversely, response bias seems to predominate in Experiment 2 (Fig. 2a). A difference in the predictability of visual stimulation might alter the balance between the effects of viewing the body on discriminability and response bias, with the more stable visual context favoring a bias effect.

### Experiment 3

#### Procedure

The procedure of Experiment 3 was the same as that of Experiment 1, except that the timing of visual stimulation was shifted to coincide with heat-pain stimulation. An inactive thermode probe placed over the foam block moved in synchrony with the probe over the participant’s hand. On each trial, either the hand or the foam block was illuminated when the thermode probe began to descend. The light turned off again after 2 s (i.e., when the probe began to retract), so that the participant saw the probe approach and contact the hand or the block. Visual presentation was randomized, as in Experiment 1, and trial and block durations were the same as in the previous experiments. Thus, Experiment 3 recapitulated Experiment 1 with the additional factor of viewing the noxious stimulation.

#### Results

Once again, the high heat-pain level was defined as the target stimulus. One participant did not have any false alarms in the hand visual condition, so a standard correction was applied to estimate the false alarm rate (Macmillan and Kaplan 1985). Mixed factor ANOVAs with the within-subjects factor ‘visual condition’ (hand or object) and the between-subjects factor ‘experiment’ (thermode probe approach visible—Experiment 3—or not visible—Experiment 1) were carried out on *d*′ scores, criterion scores, hit rates, and false alarm rates.

For discriminability (*d*′), there was an interaction between the two factors, *F*(1,30) = 6.24, *p* = .018, *η*^2^ = .169. Simple effects tests were used for follow-up comparisons. In Experiment 1, when noxious stimulation was delivered in darkness, the stimulation intensities were less discriminable when viewing the hand (*M* = 1.35, SD = 0.58) than when viewing the object (*M* = 1.66, SD = 0.73), *F*(1,30) = 5.37, *p* = .027. In contrast, when participants saw the thermode probe deliver the painful stimulus in Experiment 3, there was a nonsignificant increase in the discriminability of heat-pain intensities when viewing the hand (*M* = 1.26, SD = 0.62) compared to viewing the object (*M* = 1.09, SD = 0.66), *F*(1,30) = −1.47, *p* = .234 (Fig. 2a). Comparisons between experiments revealed lower discriminability when viewing the probe touch the object in Experiment 3 (*M* = 1.09, SD = 0.66) than when viewing the object alone in Experiment 1 (*M* = 1.66, SD = 0.73), *F*(1,30) = 5.29, *p* = .029. There was no difference in discriminability between viewing the probe touch the hand (*M* = 1.26, SD = 0.62) and viewing the hand alone (*M* = 1.35, SD = 0.58), *F*(1,30) = 0.22, *p* = .644.

There was no main effect of visual condition on *d*′, *F*(1,30) = 0.61, *p* = .441, *η*^2^ = .016, criterion, *F*(1,30) = 2.77, *p* = .106, *η*^2^ = .085, hit rates, *F*(1,30) = 1.67, *p* = .206, *η*^2^ = .051, or false alarm rates, *F*(1,30) = 2.95, *p* = .096, *η*^2^ = .084. There was also no main effect of experiment on any of these measures [*d*′: *F*(1,30) = 2.50, *p* = .124, *η*^2^ = .077; criterion: *F*(1,30) = 0.15, *p* = .704, *η*^2^ = .005; hits: *F*(1,30) = 1.49, *p* = .232, *η*^2^ = .047; false alarms: *F*(1,30) = 1.35, *p* = .255, *η*^2^ = .043]. Finally, there was no interaction between visual condition and experiment for criterion, *F*(1,30) = 0.01, *p* = .946, *η*^2^ = .0001, hits, *F*(1,30) = 1.41, *p* = .245, *η*^2^ = .042, or false alarms, *F*(1,30) = 2.19, *p* = .149, *η*^2^ = .062 (Fig. 2).

#### Discussion

The key difference between Experiments 1 and 3 was whether participants saw the thermode probe deliver the heat-pain stimulus. A between-experiments analysis revealed that the reduction in the discriminability of noxious heat intensities when viewing the hand was eliminated when participants also saw the probe deliver the noxious stimulus. In fact, there was a nonsignificant trend in the opposite direction when the probe was visible.

Importantly, seeing the approaching thermode probe in Experiment 3 did not provide any additional information about the occurrence, timing, or strength of the thermal stimulus. Participants were aware that they would feel a heat-pain stimulus on every trial, regardless of whether they saw their hand or the foam block. Moreover, the heat-pain stimulus was delivered at the same time on every trial in both experiments and was completely predictable from the offset of the LED lamp in Experiment 1. Therefore, Experiments 1 and 3 did not differ in the predictability of the noxious stimulus, but in the visual experience of watching the thermode probe contact the skin or the non-body object.

## General discussion

### Viewing the body modulates pain sensations

In Experiment 1, viewing the body reduced the discriminability of noxious heat intensities. This indicates a functional loss of information about stimulus intensity from the nociceptive system as a result of viewing the body, independent of any effect of spatial attention. Building on prior research (Longo et al. 2009, 2012; Mancini et al. 2011, 2012, 2013; Valentini et al. 2015), this experiment demonstrates that viewing the body does not merely reduce reported pain level, but inhibits the sensory processing responsible for encoding nociceptive stimulus intensity.

Previous studies suggest that visual modulation of pain occurs via connections from visual body processing areas in the extrastriate and posterior parietal cortices to areas of the pain matrix, including SI, SII, the insula, and the anterior cingulate cortex (Longo et al. 2012; Mancini et al. 2012). The results of the present study indicate that vision of the body modulates activity in regions responsible for encoding sensory/discriminative aspects of nociception. Though several nodes of the pain matrix have been implicated in processing sensory/discriminative aspects of pain (Bornhövd et al. 2002; Büchel et al. 2002; Frot et al. 2007; Hofbauer et al. 2001; Iannetti et al. 2005; Kong et al. 2006; Ohara et al. 2004; Peyron et al. 2000; Timmermann et al. 2001), some have proposed that the operculo-insular cortex has a primary role in encoding noxious stimulus intensity (Garcia-Larrea 2012a, b; Mazzola et al. 2012). In support of this claim, single pulses of transcranial magnetic stimulation (TMS) over SII, but not SI, impair performance on a pain intensity discrimination task like the one we used (Lockwood et al. 2013). Viewing the body may reduce the discriminability of noxious heat intensities through a modulatory effect of the visual body network on nociceptive processing in the operculo-insular region.

Alternatively, the effect of viewing the body on nociceptive discriminability may result from modulation of SI. At least one study found the pattern of neurophysiological activity in SI to be more consistent with pain intensity processing than the activity in SII (Timmermann et al. 2001). Moreover, responses of nociceptive neurons in monkey SI correlate with the monkey’s response time to small changes in noxious heat intensity (Kenshalo et al. 1988). Some forms of chronic pain are associated with disinhibition of the primary motor and somatosensory cortices (Eisenberg et al. 2005; Lefaucheur et al. 2006; Lenz et al. 2011; Schwenkreis et al. 2003) and with disorganization of SI somatotopic maps (Flor et al. 1995, 1997; Maihöfner et al. 2003; Tecchio et al. 2002; Wrigley et al. 2009). Modulation of SI somatotopy is therefore another possible mechanism by which viewing the body might affect the processing of pain intensity. Our study cannot distinguish between a primary somatosensory and an opercular basis for this effect.

### Viewing the body modulates pain responses

The comparison between Experiments 1 and 2 revealed that viewing the body can also bias perceived pain level irrespective of actual stimulus intensity. Interestingly, the direction of this bias was opposite to the effect found in previous studies (Longo et al. 2009, 2012; Mancini et al. 2011, 2012, 2013; Valentini et al. 2015). Participants in Experiments 1 and 2 tended to report higher levels of pain after viewing the hand. As discussed earlier, the difference in the direction of the effect on perceived pain level might be due to the kind of task. We used a forced choice discrimination task, whereas previous studies used pain detection thresholds (Mancini et al. 2011) or continuous pain rating scales (Longo et al. 2009, 2012; Mancini et al. 2012, 2013; Valentini et al. 2015).

Additionally, the timing of visual stimulation might be an important difference between present and past findings. In Experiments 1 and 2, participants had 2-s glimpses of their hand or the object that ended before contact heat-pain stimulation began. In previous studies (Longo et al. 2009, 2012; Mancini et al. 2011, 2012, 2013; Valentini et al. 2015), vision of the hand/object lasted at least 5 s and overlapped with the timing of the noxious stimulus. (In these studies, the noxious stimulus was not generally seen to contact the hand/object, either because radiant heat stimulation was used or because a mirror-reversed image of the left hand was displayed in place of the stimulated right hand. The effect of actually seeing a noxious stimulus contact the body will be discussed later.) Either the absolute duration of visual stimulation or the onset of the visual stimulus relative to the noxious stimulus might affect visual modulation of pain perception. For example, seeing one’s own hand during noxious stimulation, without seeing the noxious stimulus, might provide visual evidence that the hand is not threatened or damaged, thus biasing participants toward reporting lower heat-pain intensities. Seeing the hand before noxious stimulation would offer no such evidence. A systematic investigation of visual stimulus timing would be a valuable focus for future studies.

While our findings demonstrate that viewing the body can increase reported pain levels, other studies have shown that a complete absence of visual experience also increases perceived pain intensity. The congenitally blind have lower pain thresholds and give higher pain ratings to noxious thermal stimuli than the normally sighted and those with late-onset blindness (Slimani et al. 2013, 2014). Early visual deprivation induces structural and functional changes in neural organization which may underlie the heightened pain experiences of the congenitally blind. Alternatively, the blind may be more attentive to external threats, and this greater attention could enhance pain experiences. The latter explanation is supported by the fact that the congenitally blind also scored higher on questionnaires assessing pain vigilance (Slimani et al. 2013, 2014). Similarly, viewing the hand in Experiments 1 and 2 may have enhanced vigilance for threats to the body relative to viewing a non-body object, thereby biasing participants toward reporting higher pain levels. Viewing the noxious stimulus itself may have distinct effects, which will be discussed in the following section.

### Viewing the body under threat

Experiment 3 showed that the discriminability of noxious heat intensities is not reduced by viewing the body when the pain-inducing event is also visible. Outside the laboratory, acute pain is generally associated with a visible external event or object, making this the most naturalistic of the three experiments. Thus, while viewing the body might reduce nociceptive discriminability under certain experimental conditions, this reduction may not occur very often under everyday circumstances. Some previous studies have amalgamated the two distinct, yet interacting effects of seeing the body and seeing noxious stimulation on the body. Our results highlight the need to distinguish them.

Experiment 3 suggests that seeing a potentially harmful object approach in peripersonal space might enhance discriminability of noxious heat intensities, counteracting the effect of seeing the body itself. This enhancement might help the observer identify and avoid threats to the body. Previous studies have found that viewing an image of a needle pricking a hand on a screen over one’s own hand increases ratings of concurrently administered painful stimuli (Höfle et al. 2012, 2013). Another study found that making the arm appear red decreased pain thresholds on the arm (Martini et al. 2013). Skin redness could be perceived as a threat of bodily damage and may heighten perceived pain levels in a manner similar to viewing the approach of a threatening object. However, none of these studies measured pain perception in a way that separated perceptual effects on nociception from post-perceptual biases. Our signal detection approach allowed us to distinguish these effects. Moreover, the results of Experiment 3 suggest that viewing a threatening stimulus approach the body might boost sensory/discriminative aspects of nociception, rather than just biasing participants toward reporting higher pain levels. This explanation is called into question, however, by the observation that discriminability was only affected by whether the approaching probe was visible or not when the foam block appeared, and not when the hand appeared. If viewing a threat approach the body enhanced discriminability of noxious heat intensities, then one would expect to see a difference between viewing the hand in Experiment 1, when the probe approached in darkness, and Experiment 3, when the probe was visible.

Alternatively, viewing the foam block being touched by the probe while being touched on one’s own hand may have led the block to be ‘embodied,’ as in the rubber-hand illusion (Botvinick and Cohen 1998). Objects that do not resemble body parts are not usually embodied (Tsakiris et al. 2010, 2008; Tsakiris and Haggard 2005; but see Armel and Ramachandran 2003). Nevertheless, the salience of painful stimulation might yield a lower criterion for embodiment of a foreign object than the typical embodiment paradigm, which combines vision and innocuous touch. In Experiment 3, the appearance of touch on the foam block consistently co-occurred with a painful stimulus on the participant’s own hand, in a way that only the appearance of touch on one’s own hand normally would. The adaptive value of learning this correspondence may have outweighed the visual evidence against the foam block being a body part. We cannot form a conclusion about this from our own data, as we did not attempt to measure embodiment in this study. However, another study found a stronger rubber-hand illusion when painful tactile stimulation was used compared to innocuous tactile stimulation (Capelari et al. 2009), suggesting that noxious stimulation might strengthen the propensity to embody an external object.

### Effects of attention or intersensory conflict?

One might contend that the effect of viewing the body on pain perception is simply due to attention. However, a purely attentional account cannot explain all of our results. First, it is not clear whether the hand or the object would be more attention-grabbing. The hand might be a more interesting visual stimulus than the foam block. Alternatively, seeing the block in the location of one’s hand might increase attention because of visuo-proprioceptive incongruence. Because heightened attention tends to increase pain ratings (Arntz et al. 1991; Hodes et al. 1990; Levine et al. 1982; Miron et al. 1989) *and* improve detection of changes in noxious stimulus intensity (Bushnell et al. 1985; Miron et al. 1989), our data do not provide clear evidence for one visual condition being more attention-grabbing than the other. After viewing the hand, participants tended to report higher levels of pain, but they were also less sensitive to differences in heat-pain intensity.

Second, in Experiments 1 and 2, visual stimulation occurred prior to heat-pain stimulation. It is doubtful whether any difference in attention to the visual stimulus would have an effect on perception of the noxious stimulus, as they were presented at different times. Furthermore, in Experiments 1 and 2, the noxious stimulus was delivered in a dark and quiet environment. Though the possibility of distraction cannot be completely ruled out, these conditions should have promoted the full direction of attention toward the task-relevant noxious stimulus, regardless of the visual condition that preceded it.

Importantly, none of our experiments caused a visuo-somatosensory conflict. In Experiments 1 and 2, the noxious stimulus was administered in total darkness, following vision of the hand or the block. In Experiment 3, participants saw the thermode probe approach and contact their hand (or the foam block, which appeared in the same location). Therefore, multisensory conflict cannot readily explain the results of the experiments individually, nor the differences between viewing conditions.

### A signal detection approach to pain perception

Signal detection theory has traditionally been used to measure detection of a weak, near-threshold sensory input from noise. Nevertheless, the same formal approach can be applied to a task requiring discrimination of two suprathreshold stimuli that differ along some dimension, such as intensity. In such a case, one intensity is arbitrarily defined as the ‘target’ stimulus. This has been done with noxious heat intensities (e.g., Lockwood et al. 2013; Mancini et al. 2014), as well as suprathreshold stimuli in other sensory modalities (e.g., Ball and Sekuler 1987; Bonnel et al. 2003). Signal detection measures of sensitivity/discriminability (*d*′) and response bias (criterion) require one to calculate the proportions of ‘hits’ (target stimuli correctly identified as the target) and ‘false alarms’ (non-target stimuli incorrectly identified as the target). In the context of pain perception, which has a strong subjective component relative to other perceptual experiences, these definitions may seem problematic. For example, when participants make a ‘false alarm,’ reporting high pain when a medium-intensity heat-pain stimulus is delivered, they may indeed have felt the stimulus as highly painful. However, pain perception is, in part, underpinned by a sensory system—nociception—that can discriminate objective properties of noxious stimuli, such as location, timing, quality, and intensity (Price and Dubner 1977). Thus, when an experimental manipulation leads to lower or higher than expected reports of pain level, based on the actual stimulus intensity, we can derive information about how the underlying perceptual or post-perceptual processes have been modulated.

## Conclusions

The results of these experiments indicate that viewing the body can have two distinct effects on pain perception. First, viewing the body reduces the sensitivity of the nociceptive system to differences in the level of noxious stimulation. Second, viewing the body can bias perceived pain level regardless of actual stimulus intensity. Lastly, viewing the pain-inducing event seems to counteract the reduction in nociceptive discriminability. Together, these experiments demonstrate the importance of visual context in pain perception and highlight the need to distinguish between modulations of perceptual processing and modulations of bias.

## Footnotes

1 Note, however, that Valentini et al. (2015) only found an analgesic effect of viewing the body when the arms were crossed at the midline, and not when they were placed in a canonical position.
